# Enhanced aerodynamic and mechanical performance of double-rotor wind turbines via a one-way fluid-structure interaction analysis

**DOI:** 10.1371/journal.pone.0348271

**Published:** 2026-05-11

**Authors:** Yaru Yang, Wenhua Zhuang, Ping Yuan, Rong Yuan, Jing Yuan, Fengyun Sun, Yongxu Hu

**Affiliations:** 1 School of mechanical engineering, Chengdu University, Chengdu, Sichuan Province, China; 2 Institute of Optics and Electronics, Chinese Academy of Sciences, Chengdu, Sichuan Province, China; Isra University, JORDAN

## Abstract

Double-rotor wind turbines (DRWTs) have emerged as a promising alternative to conventional single-rotor wind turbines (SRWTs). This study proposes a novel DRWT equipped with a differential planetary gearbox and compares it with a geometrically equivalent SRWT through Computational Fluid Dynamics (CFD) and One-way Fluid-Structure Interaction (FSI) -based simulations under varying wind conditions. Dynamic meshing, six-degree-of-freedom motion, and user-defined stochastic wind loading were incorporated into the numerical framework. Compared with the SRWT, the DRWT shows 4.2% faster wake-velocity recovery in the z4 plane and a 28.35% reduction in time-varying peak blade stress compared to the equivalent ss-SRWT. Results demonstrate that the DRWT exhibits superior aerodynamic efficiency and mechanical performance, despite its more intricate structure. These findings contribute valuable insights for the operational control and structural design optimization of DRWTs.

## Introduction

The main difference between the double-rotor wind turbine (DRWT) and the traditional single-rotor wind turbine (SRWT) lies in the variation of the transmission structure and its form. Currently, research on SRWTs has focused on aspects such as improving power efficiency [1,2] and integrating with microgrids for wind speed forecasting [3]. The DRWT has become a new research hotspot in the field of wind power. Researchers have conducted multifaceted studies on the DRWT from perspectives such as structural characteristics, wind energy utilization, and performance evaluation, as well as Horizontal-axis and Vertical-axis wind turbines [4–6]. Initially, the emergence of the double-rotor wind turbine (DRWT) aimed to harness wind energy for secondary utilization. Scholars at Iowa State University [7] conducted wind tunnel experiments to study the aerodynamics and wake characteristics of a DRWT. Their findings concluded that the power generation and wind load experienced by the double-rotor wind turbine were higher compared to those of traditional single-rotor wind turbines. Vedant Kumar and Mohammad Ali Rahmatian [8,9] researched the influence of parameters such as blade geometry, rotor spacing, and other factors of the double-rotor wind turbine on its output power.

With the continuous improvement in the structural design of the DRWT and further research, Yaru Yang [10] and others have investigated the relationship between the geometric parameters and characteristic parameters of the DRWT’s rotors. Subsequently, they calculated the stress and lifespan of key components in the gearbox for both the NREL 5MW SRWT and the DRWT. The results indicated that the lifespan of the DRWT could exceed that of the traditional SRWT by more than twice [11]. Sandip A. Kale and S. N. Sapali [12] conducted evaluations on various multi-rotor wind turbine systems concerning technical performance, cost, reliability, and the safety of expected electricity generation performance.

Researchers place greater emphasis on the increase in electricity generation resulting from improvements in the aerodynamic performance of blades and mechanical performance, including multi blade VAWT [13,14]. Radu Saulescu and colleagues [15] conducted comparative analyses between counter-rotating wind turbines and wind turbines with conventional generators using simulation experiments, discovering that counter-rotating wind turbines exhibit higher electricity generation efficiency. Thomas Amoretti and other scholars [16] also studied the power generation of DRWTs and through blade element momentum (BEM) theory models, demonstrated that DRWTs generate 10.6% more electricity compared to SRWTs. Raquel Martín-San-Roman and colleagues established a free vortex filament wake module for multi-rotor wind turbines, finding that the interaction of vortex wakes between rotors can increase power generation [17].

Some scholars have conducted research on the control and operation of multi-rotor wind turbines. Rui You and others proposed a structural model for a multi-rotor medium-voltage modular wind turbine configuration that possesses advantages such as high power generation, reliability, and low transportation [18]. To address the issue of wind turbines in difficult-to-access locations, a study [19] proposed a structure with five rotor-star arrangements for multi-rotor wind turbines. Fabio Spagnolo et al. proposed an extremum-seeking controller for multi-rotor wind turbines. However, it was noted that this controller might have adverse effects on tower stresses [20]. To achieve rapid wake recovery, the proposed cyclic yaw rotor control scheme [21], increases the total power output of dual-rotor wind turbines by 15.9%.

In offshore wind farms, several scholars have focused on the research of DRWT structures. These researches mainly concentrate on the wake characteristics and control strategies of offshore DRWTs [22–24], as well as aerodynamic performance and loads [25,26]. Zhang zhihao et al. [27] proposed a wake channeling configuration that enables optimal wake recovery performance for offshore dual-rotor wind turbines (DRWTs) to increase power benefits.

The above research outcomes have provided detailed analyses of the aerodynamic performance, operational control, and other aspects concerning multi-rotor wind turbines. In aerodynamics, the studies have predominantly focused on the wake characteristics and power generation of multi-rotor wind turbines. The mechanical performance of blades during the operation of multi-rotor wind turbines is a crucial factor influencing power generation and the overall system lifespan [28].

This paper focuses on the study of a novel DRWT with differential planetary gearbox. Based on the existing data, the finite element models for the blades of DRWT and ss-SRWT are built. Using the fluid-structure interaction (FSI) module in ANSYS, simulation experiments are conducted under random wind speeds for both the DRWT and the Single-Rotor Wind Turbine with the same power and scale (ss-SRWT). To enhance the fidelity of the simulation experiments and better replicate real-world operating conditions, this study incorporates dynamic mesh generation and six degrees of freedom (Six-DOF) capabilities within the Fluid-Structure Interaction (FSI) framework. Additionally, a User-Defined Function (UDF) is developed to model stochastic wind conditions. By comparing the experimental results, the advantages of the DRWT in aerodynamics and mechanical performance are validated, providing theoretical basis for the operation control and blade structural design of DRWT.

## Models

### Geometric model of DRWT and ss-SRWT

**Fig 1:**
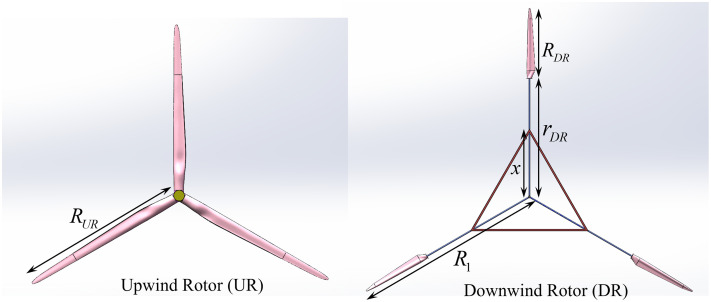
Geometric Model of UR and DR.

The DRWT (Dual-Rotor Wind Turbine) comprises two rotors installed on either side of the nacelle, which are operated through a differential planetary speed-increasing system to drive the generator. During the operation of the DRWT, the rotor blade positioned on the windward side is referred to as the Upwind Rotor (UR), while the blade on the leeward side of the nacelle is termed the Downwind Rotor (DR). A schematic diagram of the UR and DR structure is shown in Fig 1.

The Upwind Rotor (UR) of the DRWT utilizes the structure of a conventional horizontal-axis wind turbine—the NREL 5MW wind turbine, as presented in Table 1 [29].

**Table 1:** Relevant parameters of NREL 5MW wind turbine.

Rated Power	5 MW
Rotor Diameter	126m
Rated Wind Speed	11.4m/s
Rated Rotor Speed	12.1rpm
Rated Tip Speed	80m/s
Hub Diameter	3m

When determining the geometric structure of DR, the DR blades are mounted using a triangular support structure on the outer periphery of the UR blade’s swept surface to minimize the overlap between the swept surfaces of UR and DR blades. In the Fig 1, $R_{\text{1}}$ represents the distance from the tip of the DR blades to the center of rotation, $r_{DR}$ represents the distance from the root of the DR blades to the center of rotation, and x represents the distance from the fixed point of the triangular support on the supporting rod to the center of rotation.

The power absorbed by the wind rotor can be calculated by formula $E\text{=}\frac{\text{1}}{\text{2}}C\rho Av^{\text{3}}$, and the power absorbed by the double rotor can be calculated as follows:


$$E_{UR}=\frac{\text{1}}{\text{2}}C\rho \pi A_{UR}\overset{\text{3}}{\underset{UR}{v}}=\frac{\text{1}}{\text{2}}C\rho \overset{\text{3}}{\underset{UR}{v}}\pi \overset{\text{2}}{\underset{UR}{R}}$$ (1)

$$E_{DR}=\frac{\text{1}}{\text{2}}C\rho \pi A_{DR}\overset{\text{3}}{\underset{DR}{v}}=\frac{\text{1}}{\text{2}}C\rho \overset{\text{3}}{\underset{DR}{v}}\pi \text{(}\overset{\text{2}}{\underset{\text{1}}{R}}-\overset{\text{2}}{\underset{DR}{r}})$$ (2)

then,


$$\phi =\frac{E_{UR}}{E_{DR}}=\frac{\frac{\text{1}}{\text{2}}C\rho \overset{\text{3}}{\underset{UR}{v}}\pi \overset{\text{2}}{\underset{UR}{R}}}{\frac{\text{1}}{\text{2}}C\rho \overset{\text{3}}{\underset{DR}{v}}\pi (\overset{\text{2}}{\underset{\text{1}}{R}}-\overset{\text{2}}{\underset{DR}{r}})}=\frac{\overset{\text{3}}{\underset{UR}{v}}\overset{\text{2}}{\underset{UR}{R}}}{\overset{\text{3}}{\underset{DR}{v}}(\overset{\text{2}}{\underset{\text{1}}{R}}-\overset{\text{2}}{\underset{DR}{r}})}$$ (3)

Physical meanings of each parameter in the above equations (1)–(3) are as follows:

E: The energy absorbed by the turbine blades from the wind, which is the power generated by the blades.

$E_{UR}\text{,}E_{DR}$: The power generated by the UR and DR.

C: The coefficient of wind energy utilization.

ρ: Air density.

A: The swept area of the blades, $A\text{=}\pi R^{\text{2}}$, where R represents the blade length.

$A_{UR}\text{,}A_{DR}$: The swept area of the UR blades and the DR blades.

$R_{UR}\text{,}R_{DR}$: The length of the UR blade and the DR blade.

$v_{UR}\text{,}v_{DR}$: The wind speed over the swept area of the UR and DR.

**Fig 2:**
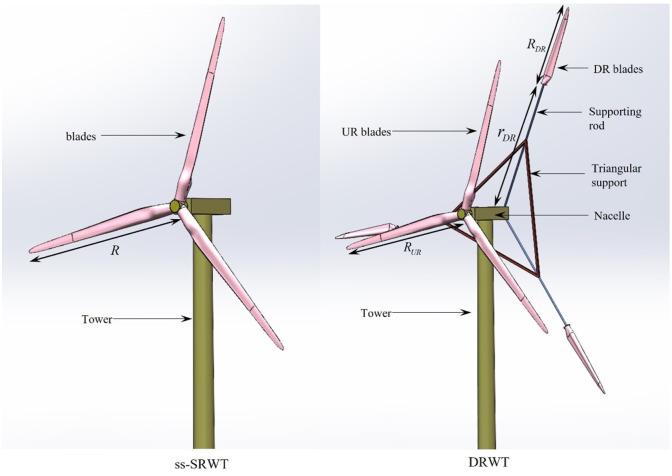
Geometric model of DRWT and ss-SRWT.

Based on the author’s previous study on the parameters and relationships between the two rotors of the DRWT in reference [10], this paper selects a power ratio ϕ of 1 between UR and DR. The structural parameters for the DR support are taken from reference [10], setting $x=0.6r_{DR}$ as shown in Fig 1. The parameters for UR and DR are shown in Table 2. The power of the ss-SRWT is equivalent to that of the DRWT, with a blade length of 126 meters. The blade structure also follows that of the NREL 5MW wind turbine, and the geometric model of the DRWT and ss-SRWT is illustrated in Fig 2.

**Table 2:** Geometric Parameters of UR and DR.

Item	Data	Units
Length of the UR blade ($R_{UR}$)	63	m
Hub diameter of the UR	3	m
Length of the DR blade ($R_{DR}$)	25	m
Hub diameter of the DR	4	m
Length of the connecting rod of the triangular support	54	m
Cross-sectional area of the triangular support	0.7	$m^{\text{2}}$
Length of the blade support rod for the DR ($r_{DR}$)	52	m
Cross-sectional area of the blade support rod for the DR	0.5	$m^{\text{2}}$

### Calculation model for the mechanical performance

The incident wind speed acting on the wind turbine’s blades generates aerodynamic forces, thereby driving the blades to rotate. Simultaneously, this aerodynamic force directly affects the blades, causing deformation.

**Fig 3:**
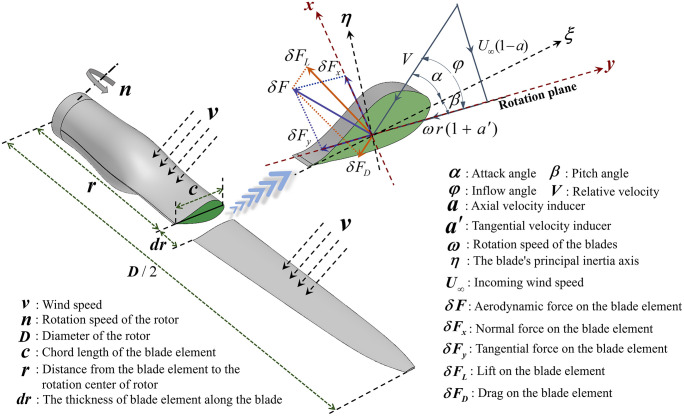
Geometric parameters and aerodynamic force of the blade elements.

The computation of aerodynamic forces acting on wind turbine blades is highly complex. It involves dividing the blade along its span into numerous elemental sections (blade elements). Considering the entire blade as composed of multiple blade elements assumes independent forces acting on each element. This simplification transforms the three-dimensional model of the blade into a two-dimensional model. Consequently, based on the aerodynamic characteristics of airfoils, the aerodynamic forces acting on these blade elements can be calculated [30], as illustrated in Fig 3.

The relationship between the airflow velocity triangles in Fig 3 is as follows.


$$V=\sqrt{\overset{\text{2}}{\underset{\infty }{U}}(1-a{)}^{\text{2}}+\omega^{\text{2}}r^{\text{2}}(1+a^{\prime }{)}^{\text{2}}}$$ (4)

$$sin\;\varphi =\frac{U_{\infty }(1-a)}{V}$$ (5)

$$cos\;\varphi =\frac{r\omega (1+a^{\prime })}{V}$$ (6)

β=φ−α (7)

According to the aerodynamic characteristics of the 2D airfoil [31], the aerodynamic lift $\delta F_{L}$ and drag $\delta F_{D}$ experienced by the blade element are calculated as follows (ρ is the air density).


$$\delta F_{L}=\frac{\text{1}}{\text{2}}C_{L}\rho V^{\text{2}}c\cdot dr$$ (8)

$$\delta F_{D}=\frac{\text{1}}{\text{2}}C_{D}\rho V^{\text{2}}c\cdot dr$$ (9)

According to the geometric relationships depicted in Fig 3, the aerodynamic lift $\delta F_{L}$ and drag $\delta F_{D}$ on the blade element can be decomposed into the flow direction of the airstream (axial) and the blade’s rotation direction (tangential). Therefore, the axial and tangential forces, $\delta F_{x}$ and $\delta F_{y}$, on the blade element can be derived as follows.


$$\delta F_{x}=\delta F_{L}cos\;\varphi +\delta F_{D}\;sin\;\varphi =\frac{\text{1}}{\text{2}}C_{x}\rho V^{\text{2}}c\cdot dr$$ (10)

$$\delta F_{y}=\delta F_{L}sin\;\varphi -\delta F_{D}\;cos\;\varphi =\frac{\text{1}}{\text{2}}C_{y}\rho V^{\text{2}}c\cdot dr$$ (11)

In Equations (10) and (11), $C_{x}$ and $C_{y}$ denote the axial and tangential force coefficients, respectively.


$$C_{x}=C_{L}cos\varphi +C_{D}\;sin\varphi$$ (12)

$$C_{y}=C_{L}sin\varphi -C_{D}\;cos\varphi$$ (13)

By substituting the geometric parameters of the UR and DR into Equations (8)–(11) and integrating along the blade span, the aerodynamic loads on both rotors can be obtained.


$$F_{UR}=\frac{\text{1}}{\text{2}}\rho V^{\text{2}}\int_{\text{0}}^{R_{UR}}c(\overset{\text{2}}{\underset{L}{C}}+\overset{\text{2}}{\underset{D}{C}}{)}^{1/2}dr$$ (14)

$$F_{DR}=\frac{\text{1}}{\text{2}}\rho V^{\text{2}}\int_{0.89R_{UR}}^{1.22R_{UR}}c(\overset{\text{2}}{\underset{L}{C}}+\overset{\text{2}}{\underset{D}{C}}{)}^{1/2}dr$$ (15)

The blade model is based on the NREL 5 MW wind turbine, for which the airfoil geometry and material properties are well documented. As a slender structure, the blade is modeled as a cantilever beam [32,33].

The normal stress on the blade section comprises two components: the stress induced by the centrifugal force $\sigma_{c}$ and the stress induced by the bending moment $\sigma_{b}$.


$$\sigma =\sigma_{c}+\sigma_{b}$$ (16)

Where, $\sigma_{c}$ and $\sigma_{b}$ can be calculated respectively using formulas $\sigma_{c}=\frac{\rho_{\text{0}}\omega^{\text{2}}}{F\left(r\right)}\int_{r}^{R}r_{\text{0}}F_{\text{0}}dr_{\text{0}}$ and $\sigma_{b}=\frac{M_{\eta }}{J_{\eta }}\xi$, where $\rho_{\text{0}}$ stands for the density of the blade material, ω denotes the blade rotation speed, $F_{\text{0}}$ represents the equivalent area of the airfoil section, $r_{\text{0}}$ is the integral variable, $M_{\eta }$ denotes the bending moment on the blade’s principal inertia axis η, $J_{\eta }$ signifies the minimum principal inertia, as illustrated in Fig 3.

The deflection of a blade element at any position r along the blade in the x,y -direction can be calculated using the following two equations.


$$f_{x}=\int_{R_{\text{0}}}^{r}\frac{M_{\eta }}{EJ_{\eta }}(R_{tip}-r)sin\;\beta dr$$ (17)

$$f_{y}=\int_{R_{\text{0}}}^{r}\frac{M_{\eta }}{EJ_{\eta }}(R_{tip}-r)cos\;\beta dr$$ (18)

Where, $R_{\text{0}}$ stands for the radius at the root of the blade, and $R_{tip}$ represents the radius at the blade tip.

The twist angle of the blade can be calculated using the torsion equation for thin-walled members.


$$\Delta \varphi =\int \frac{M_{k}}{GJ_{k}}dr$$ (19)

Where, $M_{k}$ stands for the torsional moment, G represents the shear modulus, and $J_{k}$ is the torsional inertia.

## Methodology and experiments

There are various numerical simulation methods available for assessing the operation of wind turbines. In the present study, the fluid–structure interaction framework is implemented as a one-way coupling strategy, in which the aerodynamic loads obtained from the CFD simulations are transferred to the structural model, while the structural deformation is not fed back to the flow solver.

### CFD model

The linear velocity of the tip rotation of DRWT does not exceed 80 m/s, corresponding to the Mach number Ma=v/c≤80/340=0.24 (where v represents the tip linear velocity and c represents the speed of sound). Therefore, when studying the flow field of DRWT, air can be considered incompressible, with its governing equations being the incompressible Navier-Stokes (N-S) equations [34], as shown below.


$$\frac{\partial }{\partial t}\int_{\Omega }\vec{W}d\Omega +\oint_{\partial \Omega }({\vec{F}}_{C}-{\vec{F}}_{V})d\vec{S}=0$$ (20)

In the equation, ∂Ω represents the boundary of the control volume, $\vec{W}$ represents the conserved variable, $\vec{F_{C}}$ and $\vec{F_{V}}$ respectively represent the convective flux vector and the viscous flux vector.


$$\vec{W}=\left[\begin{array}{c} @l@\rho \\ \rho u\\ \rho v\\ \rho w \end{array}\right],{\vec{F}}_{C}=\left[\begin{array}{c} @l@\rho V\\ \rho uV+n_{x}p\\ \rho vV+n_{y}p\\ \rho wV+n_{z}p \end{array}\right],{\vec{F}}_{V}=\left[\begin{array}{c} @l@0\\ n_{x}\tau_{xx}+n_{y}\tau_{xy}+n_{z}\tau_{xz}\\ n_{x}\tau_{yx}+n_{y}\tau_{yy}+n_{z}\tau_{yz}\\ n_{x}\tau_{zx}+n_{y}\tau_{zy}+n_{z}\tau_{zz} \end{array}\right]$$ (21)

In the equation, ρ represents air density, p represents pressure, $n_{x},n_{y},n_{z}$ represent the three components of the unit outward normal vector of the surface of control volume Ω. u,v,w represent the three components of velocity $\vec{u}$. The inverse velocity V can be expressed as follows.


$$V=\vec{u}\cdot \vec{n}=n_{x}u+n_{y}v+n_{z}w$$ (22)

$\tau_{ij}$ represents the tensor of shear stress, including the effects of laminar viscosity and turbulent viscosity, and can be specifically expressed as follows.


$$\begin{array}{c} \tau_{xx}=\frac{\text{2}}{\text{3}}\mu (\frac{\partial u}{\partial x}+\frac{\partial v}{\partial y}+\frac{\partial w}{\partial z})+2\mu \frac{\partial u}{\partial x},\tau_{xy}=\tau_{yx}=\mu (\frac{\partial u}{\partial y}+\frac{\partial v}{\partial x})\\ \tau_{yy}=\frac{\text{2}}{\text{3}}\mu (\frac{\partial u}{\partial x}+\frac{\partial v}{\partial y}+\frac{\partial w}{\partial z})+2\mu \frac{\partial v}{\partial y},\tau_{xz}=\tau_{zx}=\mu (\frac{\partial u}{\partial z}+\frac{\partial w}{\partial x})\\ \tau_{zz}=\frac{\text{2}}{\text{3}}\mu (\frac{\partial u}{\partial x}+\frac{\partial v}{\partial y}+\frac{\partial w}{\partial z})+2\mu \frac{\partial w}{\partial z},\tau_{yz}=\tau_{zy}=\mu (\frac{\partial v}{\partial z}+\frac{\partial w}{\partial y}) \end{array}$$ (23)

In the equation, μ represents the viscosity coefficient, which is the sum of the laminar viscosity coefficient $\mu_{L}$ and the turbulent viscosity coefficient $\mu_{T}$, i.e., $\mu =\mu_{L}+\mu_{T}$.

**Fig 4:**
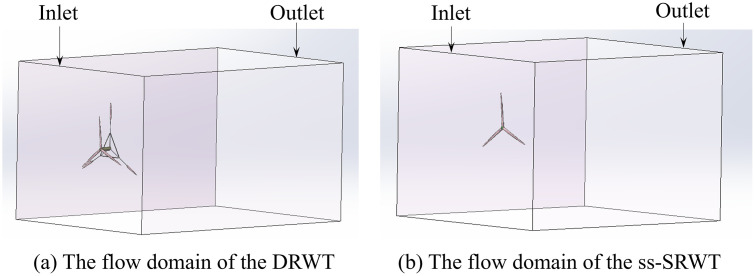
The computational flow domain and boundary conditions for the DRWT and ss-SRWT models. The setup includes a velocity inlet (stochastic wind profile, 1D upstream) and a pressure outlet (zero-gauge pressure, 6D/5.5D downstream). Symmetry conditions are applied to the lateral and top/bottom boundaries.

CFD numerical simulations of wind turbines require the establishment of a flow field domain [35], followed by using Fluent to analyze and compute the fluid motion processes within the flow field domain. The fluid domain dimensions for the DRWT and ss-SRWT are 6D × 3D × 3D and 5.5D × 3D × 3D, respectively, as shown in Fig 4. To prevent artificial acceleration and blockage effects, the computational domain was carefully dimensioned. The distance from the rotor center to the velocity inlet was set to 1D upstream, while the pressure outlet was positioned 6D/5.5D downstream for DRWT and ss-SRWT respectively to allow for full wake development and pressure recovery. The lateral and top/bottom boundaries were placed at a distance of 1.5D from the rotor axis and were assigned symmetry (or free-slip) boundary conditions.

**Fig 5:**
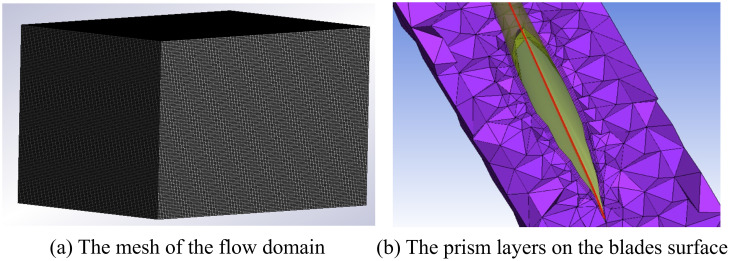
The mesh of the flow domain and the prism layers on the surface of the blades.

The computational fluid domain (as shown in Fig 4) was discretized using an unstructured polyhedral/tetrahedral mesh with local refinements in the wake regions and dynamic mesh zones. To accurately capture the boundary layer flow and flow separation near the blade surfaces, 5 prism inflation layers were generated around the DRWT and ss-SRWT blades (as shown in Fig 5). The first layer thickness was strictly controlled to ensure that the non-dimensional wall distance (y+) remained within an optimal range. Specifically, the maximum y+ value on the blade surfaces was maintained below 3.0, with an average y+ distribution of approximately 1.5. This fine resolution satisfies the near-wall treatment requirements of the turbulence model k−ε used in this study.

To ensure the robustness of the numerical results, a mesh independence study was conducted on the DRWT model prior to the formal simulations. Three different mesh schemes (Coarse: 18.2 million, Medium: 30.9 million, Fine: 45.1 million) were generated by refining the grid size in the rotor rotation region and the blade surface layers. The average torque on the Upwind Rotor (UR) under a steady rated wind speed of 11.4 m/s was selected as the key indicator for comparison. As shown in Table 3, the relative difference in average torque between the medium and fine meshes was less than 1.5%, while the computational cost for the fine mesh increased by approximately 48%. Therefore, considering the balance between computational accuracy and efficiency, the medium mesh scheme (30,903,797 cells) was selected for all subsequent FSI simulations. A similar procedure was followed for the ss-SRWT model.

**Table 3:** Mesh Independence Study for the DRWT Model.

Mesh Scheme	Number of Cells (Million)	Average UR Torque (kN·m)	Relative Difference (%)
Coarse	18.2	198.5	--
Medium	30.9	207.2	4.38% (vs. Coarse)
Fine	45.1	209.8	1.25% (vs. Medium)

### Set up and initial conditions

After completing the pre-processing, some parameters and initial conditions need to be set. In Fluent, the transient pressure-based solver and the k−ε turbulence model are selected. The standard k−ε model was chosen due to its robustness, computational efficiency, and proven effectiveness in simulating the far-wake and overall performance characteristics of wind turbines at high Reynolds numbers. While models like k−ω SST may offer higher fidelity in resolving boundary layer separation near the blade surface, the primary focus of this study is on the overall aerodynamic loads for FSI analysis and the far-field wake structure. For these objectives, the standard k−ε model provides a well-established balance between accuracy and computational cost for industrial-scale aerodynamic simulations. Inlet and outlet boundaries are defined accordingly as “Inlet” and “Outlet”. At the velocity inlet, the turbulence intensity (TI) was specified as 5% to represent typical atmospheric boundary layer conditions. The turbulent viscosity ratio is 10 to accurately define the incoming turbulent dissipation rate.

To ensure a rigorous and fair comparative analysis, the equivalent single-rotor wind turbine (ss-SRWT) was strictly conFigd to match the Double-Rotor Wind Turbine (DRWT) in terms of total rated power and overall scale. Specifically, the total swept area of the DRWT (the sum of the upwind and downwind rotors) was designed to equal the swept area of the ss-SRWT. Consequently, as noted in the stress analysis, the individual blade lengths of the DRWT are shorter than those of the ss-SRWT. The mass and moment of inertia for the blades of both models were calculated and scaled proportionally based on their identical material density and respective geometric dimensions.

Furthermore, to isolate the aerodynamic and mechanical effects of the rotor configurations, both the DRWT and ss-SRWT models share identical hub heights, nacelle geometries, and tower support structures. In the Fluid-Structure Interaction (FSI) simulations, consistent boundary conditions were strictly applied to both models, including identical stochastic inlet wind velocity profiles, zero-pressure outlet conditions, and rigid fixed-support boundary conditions at the base of the towers.

When conducting the fluid simulation using dynamic mesh technology, it is necessary to activate the dynamic mesh module in Fluent, set the grid updating method to “Smoothing and Remeshing”, and activate the Six Degrees of Freedom (Six DOF) feature. Furthermore, a time-step sensitivity analysis was performed to ensure the temporal accuracy of the transient simulation. Three-time steps (Δt=0.01s,0.005s,0.002s) were tested. The analysis revealed that the difference in the predicted peak blade root bending moment between Δt=0.005s and Δt=0.002s was negligible (< 2%). Thus, a time step of 0.005s was chosen to maintain a Courant number (CFL) generally below 2 in the rotating domain, which is sufficient to capture the key transient aerodynamic phenomena without incurring excessive computational expense.

To accurately capture the dynamic interaction between the fluid flow and the turbine structure, a two-way implicit coupling scheme was adopted using the ANSYS System Coupling module. The time steps for both the CFD (FLUENT) and structural (Mechanical) solvers were strictly synchronized at 0.005 s to align the physical time domains. Within each physical time step, a maximum of five coupling iterations (coupling frequency) were executed to exchange aerodynamic forces and nodal displacements iteratively until convergence was reached. To suppress numerical oscillations and ensure stable data transfer across the fluid-structure interface, an under-relaxation strategy was implemented, setting a constant under-relaxation factor of 0.75 for the displacement data transfer. The convergence criterion for the FSI coupling loop was defined by a Root Mean Square (RMS) residual target of $10^{-4}$ for both force and displacement transfers.

**Fig 6:**
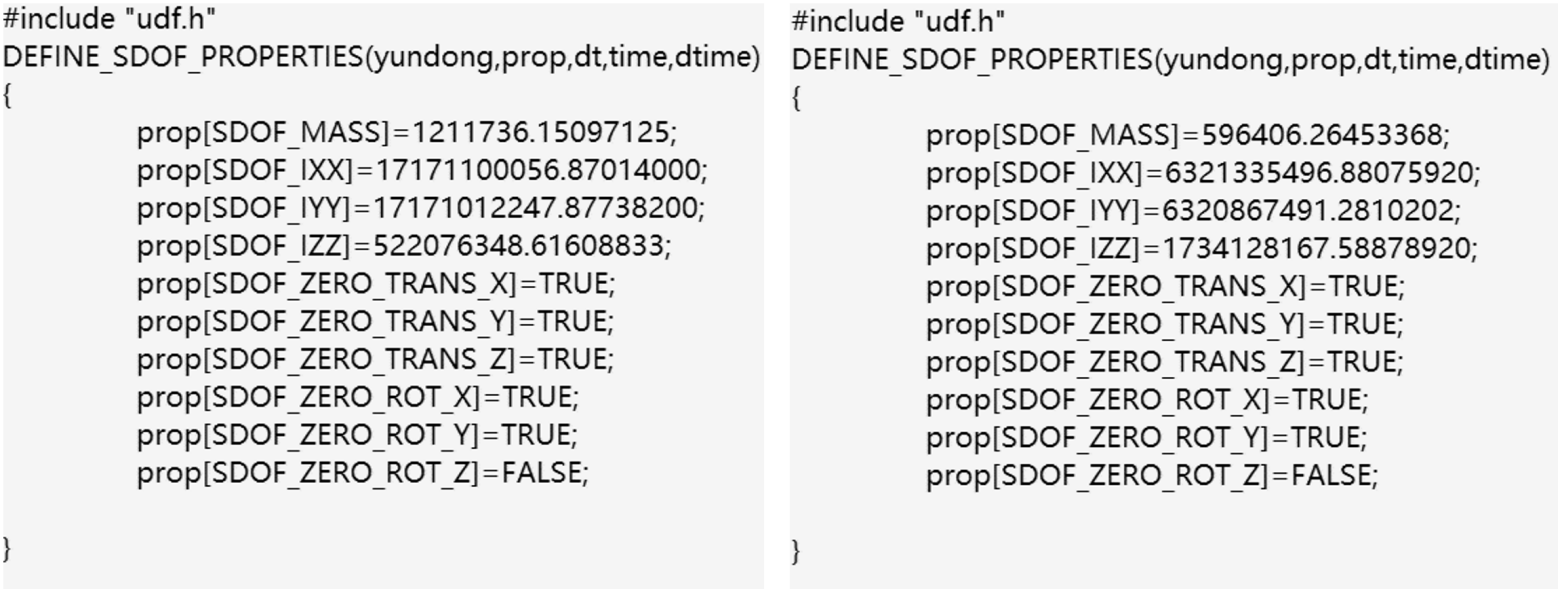
The UDF code for defining the motion.

**Fig 7:**
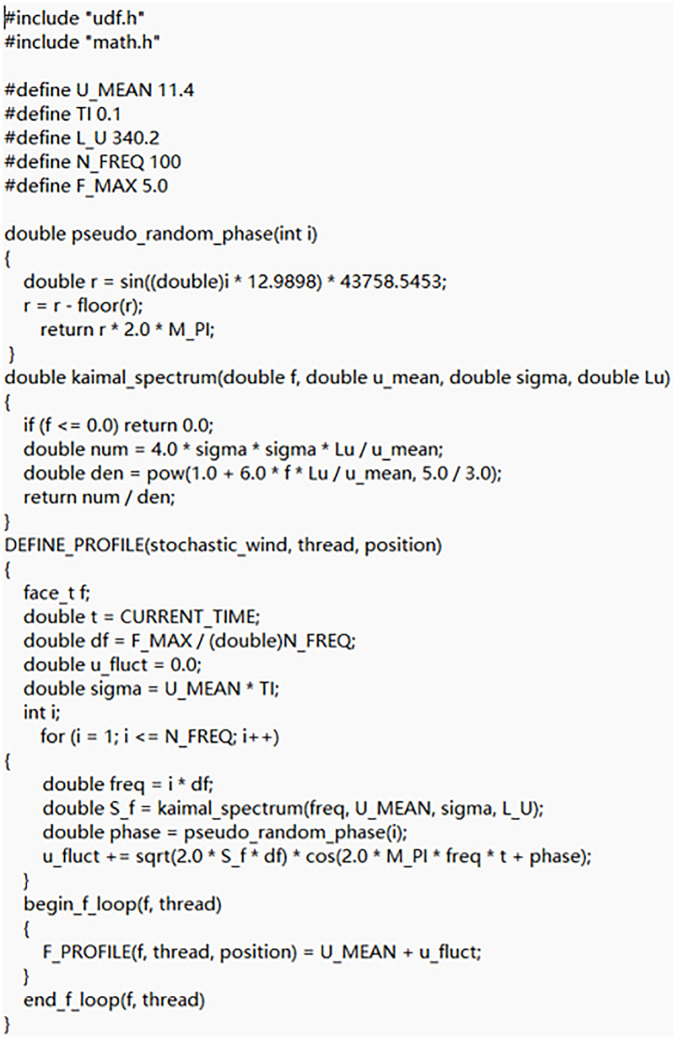
The UDF code for defining stochastic wind velocity.

It is necessary to compile UDF program code to specify the motion mode of the grid in the motion region. To capture the passive rotation of the rotors driven by aerodynamic forces, a Six-DOF UDF was compiled to define the mass and moments of inertia of the DRWT, strictly limiting the motion to rotational degrees of freedom about the z-axis (as shown in Fig 6). Furthermore, to simulate realistic real-world operating conditions, the stochastic wind velocity profile at the inlet boundary was generated using a separate UDF (the code was shown in Fig 7). Instead of using artificial random numbers, the turbulence velocity fluctuations were generated based on the Harmonic Superposition Method [36] utilizing the Kaimal turbulence spectrum (IEC 61400−1 standard) [37]. By discretizing the Kaimal power spectral density function and superimposing multiple harmonic cosine waves [38] with uniformly distributed random phase angles ϕ∈(0,2π), the UDF ensures that the temporal frequency characteristics and spectral energy distribution of the simulated inlet wind align with realistic atmospheric boundary layer turbulence.

## Results

### The results of Fluent

The paper established four planes located behind the swept area of the rotors in the flow domain of both DRWT and ss-SRWT, specifically at axial positions of 0.2D($z_{\text{1}}$)、0.4D($z_{\text{2}}$)、0.6D($z_{\text{3}}$)、0.8D($z_{\text{4}}$) along the z-axis in the coordinate system. Using computational results of Fluent, the velocity and pressure distribution characteristics on these planes were analyzed. Additionally, since the aerodynamic forces on the surface of the blades during operation have a crucial impact on the mechanical performance of the blades (including stress, deformation, etc.), this paper analyzed the aerodynamic forces (including normal aerodynamic force and tangential wall shear force) on the surfaces of the rotors of both DRWT and ss-SRWT.

### The distribution of wind speed

**Fig 8:**
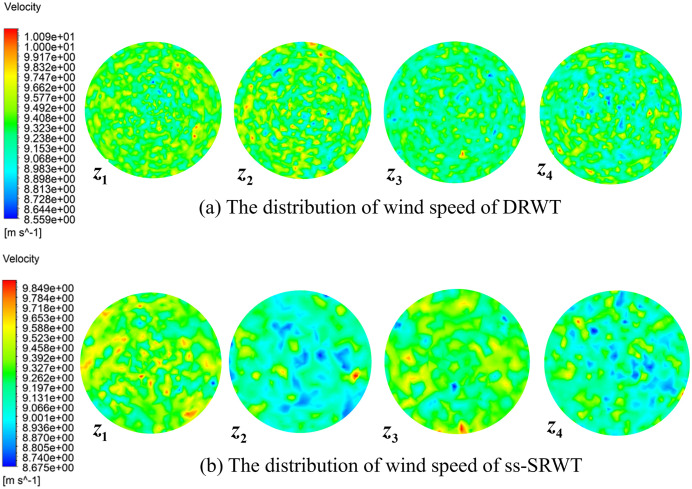
Contours of the wind velocity distribution at four axial positions (0.2D, 0.4D, 0.6D, 0.8D) in the near wake region. Simulation parameters: Rated mean wind speed U = 11.4 m/s, approximate Reynolds number $Re=5\times 10^{\text{6}}$, and transient time step Δt=0.005s.

Fig 8 illustrates the contours of the wind velocity distribution at four axial positions in the near wake region behind the swept areas. Overall, for both DRWT and ss-SRWT, the wind velocity in the Figs exhibits a multi-region vortex-like distribution. This is related to the complex vortex system formed at the trailing edge of the blades when the rotor rotates. The wake vortex system of the entire rotor can be visualized as being composed of attached vortices from the blades, free vortices in the helical shape of the blade tips, and central vortices from the blade roots. Compared with the ss-SRWT, the DRWT wake contains a larger number of localized small-scale vortical structures, which are generated by the interaction between the upwind-rotor wake and the inflow encountered by the downwind rotor. This is because both rotors, UR and DR, of DRWT can generate wake vortex systems, and the interaction between the vortex systems of the two rotors complicates the wake structure more than in the ss-SRWT.

**Fig 9:**
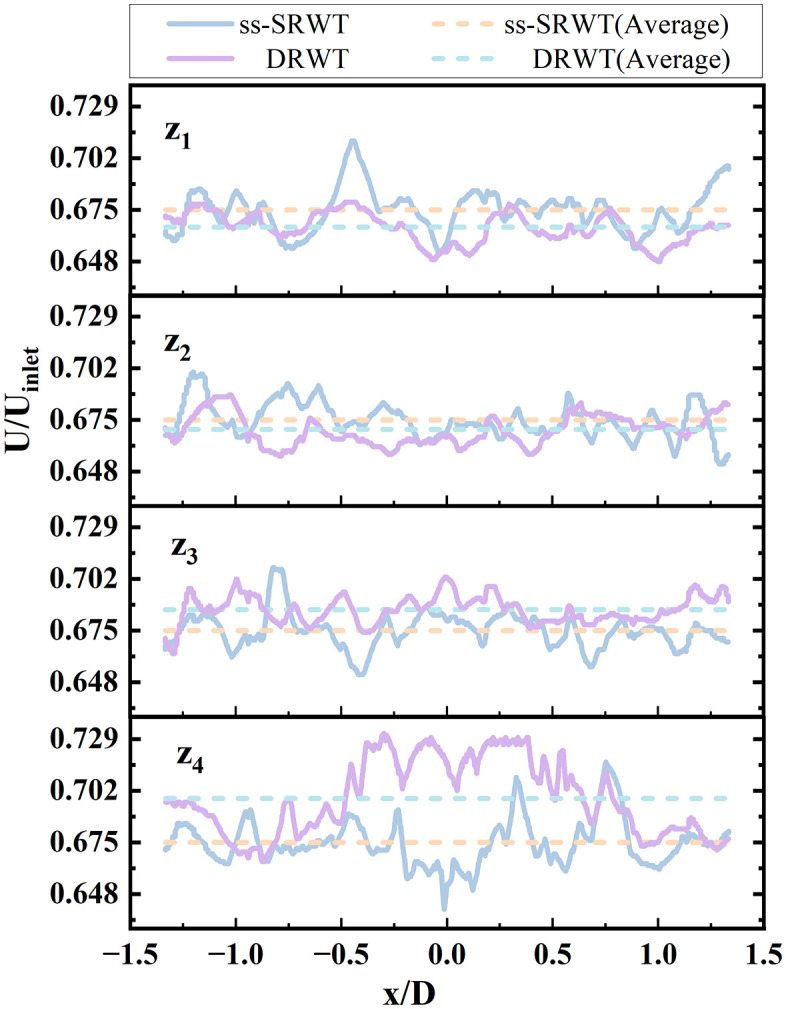
Normalized wind speed distributions of DRWT and ss-SRWT.

To quantitatively evaluate the wake recovery performance, the normalized velocity magnitude $U/U_{inlet}$ was calculated at the defined axial planes (as shown in Fig 9). Statistical analysis shows that at the plane $z_{\text{4}}$ position, the average normalized velocity of the DRWT wake region is 0.698, whereas it is only 0.675 for the ss-SRWT. This represents a 3.5% improvement in wake velocity recovery. Furthermore, the standard deviation of the velocity distribution (σ) across the swept area was lower for the DRWT ($\sigma_{DRWT}=0.095$) compared to the ss-SRWT ($\sigma_{S-DRWT}=0.133$), indicating a more uniform flow field which contributes to reduced aerodynamic load fluctuations.

### The aerodynamic performance

**Fig 10:**
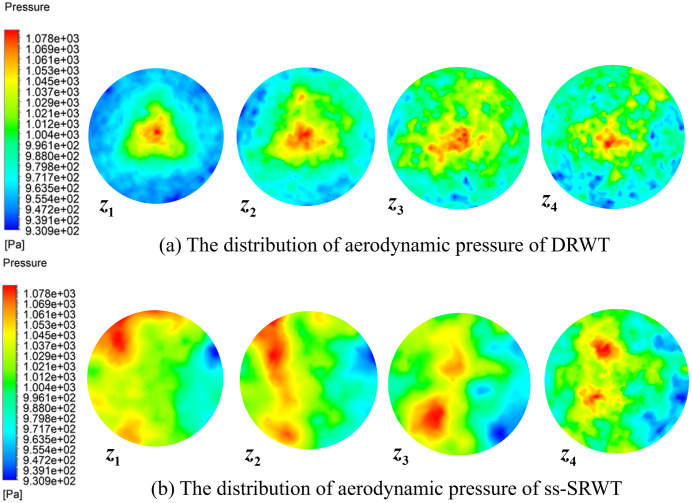
The contour of aerodynamic pressure.

Fig 10(a), (b) respectively depict the contours of the aerodynamic pressure distribution on the four planes $z_{\text{1}}\sim \;z_{\text{4}}$ at axial positions behind the swept areas of DRWT and ss-SRWT. The pressure in the near wake region of both DRWT and ss-SRWT gradually increases with the increasing of the axial distance. When positioned at the same axial location behind the rotors along the z-axis, the pressure in DRWT is higher than that in ss-SRWT. Additionally, the pressure distribution on the axial planes of DRWT exhibits a decreasing trend from the center towards the periphery. The region of higher aerodynamic pressure on the axial planes of ss-SRWT gradually decrease with the increasing of the axial distance. These differences are related to the structural characteristics of the two turbines and are also associated with the different dynamic performance caused by structural differences during operation.

**Fig 11:**
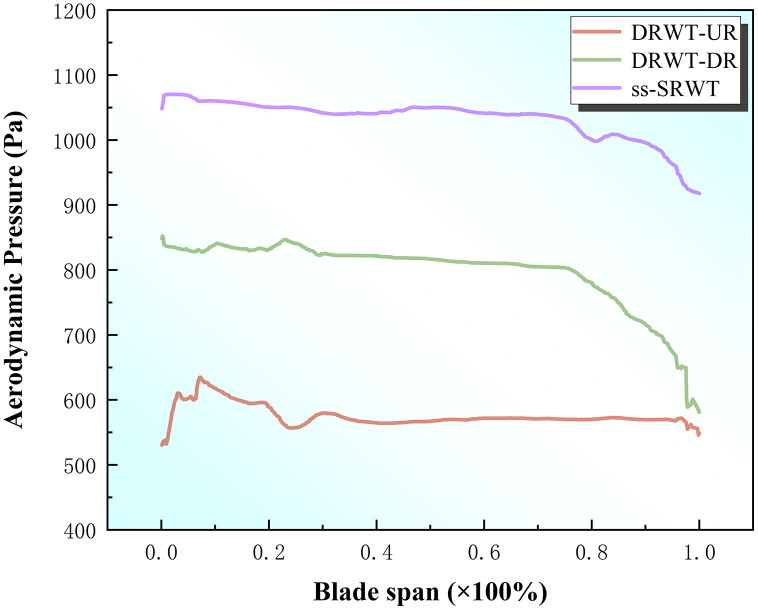
The curves of aerodynamic pressure of single blade.

Fig 11 depicts the distribution of aerodynamic pressure on the blades of DRWT and ss-SRWT along the spanwise direction. From the Fig, it could be observed that the aerodynamic pressure on the blades remains relatively consistent along the span from the root to the tip, with a slight decrease. The maximum aerodynamic pressure on the blades of ss-SRWT is approximately 1150 Pa. Following that, the aerodynamic pressure on the blades of DRWT-DR is approximately 600–850 Pa, while the aerodynamic pressure on the blades of DRWT-UR is the lowest at around 500 Pa.

**Fig 12:**
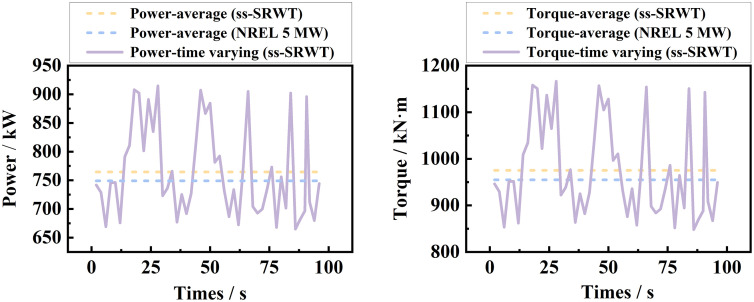
Power and torque lines of the ss-SRWT and the NREL 5MW wind turbine.

Because the DRWT featuring a differential planetary gearbox is a novel configuration, direct experimental data for the entire system is currently unavailable. Therefore, to validate our CFD and FSI framework, we utilized our geometrically equivalent Single-Rotor Wind Turbine (ss-SRWT) model and compared its aerodynamic power and torque against the widely recognized NREL 5MW reference turbine experimental data (when the wind speed is 6 m/s). We plotted these data as shown in Fig 12. In the Fig, the differences in average power and average torque (the blue and orange dashed lines) between the ss-SRWT and the NREL 5MW wind turbine are only 2.08% and 2.11%, respectively, both not exceeding 2.2%. This demonstrates that the fluid-structure interaction numerical method employed in this paper is effective and reliable.

### The normal force and tangential force of blades

**Fig 13:**
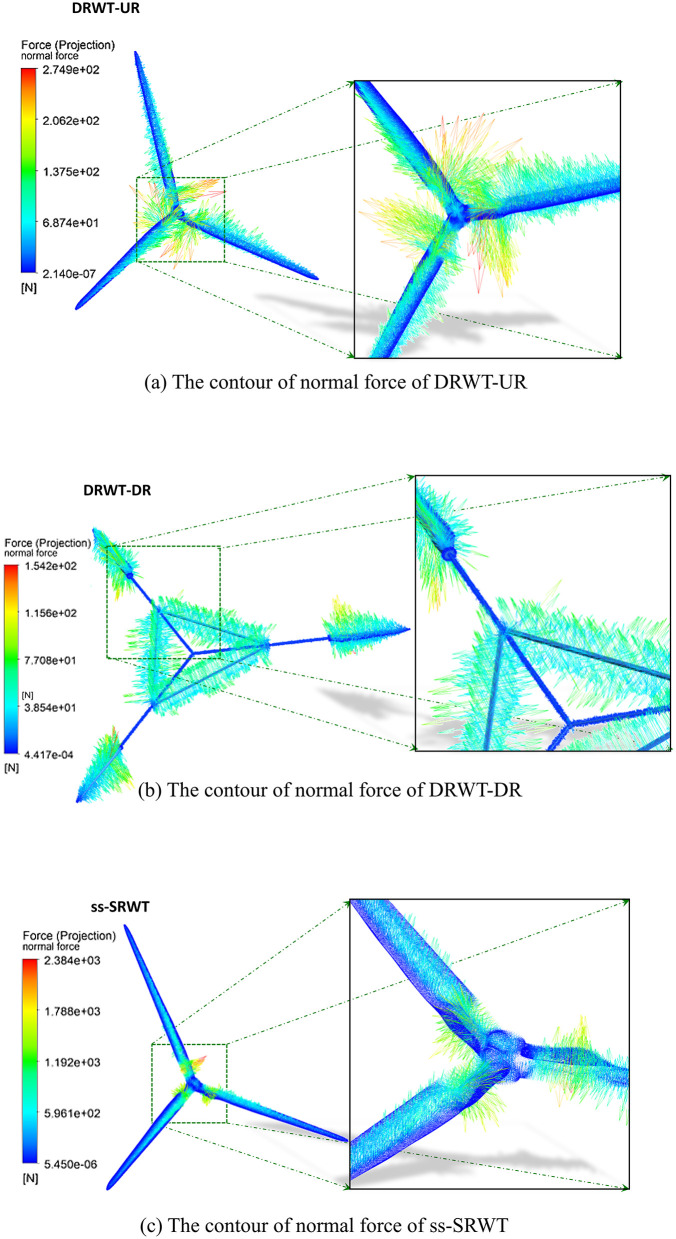
The vector contour of normal force. (Simulation parameters: U = 11.4 m/s, $Re=5\times 10^{6}$, time step Δt=0.005s).

The vector contours of the normal force on the surfaces of the blades of DRWT and ss-SRWT are shown in Fig 13. The normal forces acting on the blades of ss-SRWT are mostly concentrated around 600 N, with a larger normal force reaching above 2000 N at the blade root. The normal forces on the blades of DRWT-UR are mostly around 70 N, with the largest force at the blade root not exceeding 300 N. Conversely, the normal forces on the blades of DRWT-DR are smaller, around 40 N, and the maximum force near the connection between the blades and the support rods is only about 150 N. It can be observed that the normal forces on the surfaces of the blades of both rotors in DRWT is significantly lower than that of ss-SRWT. This is directly correlated with the alleviation of stress concentration on the blades of the DRWT, as discussed in Section “The aerodynamic performance”.

**Fig 14:**
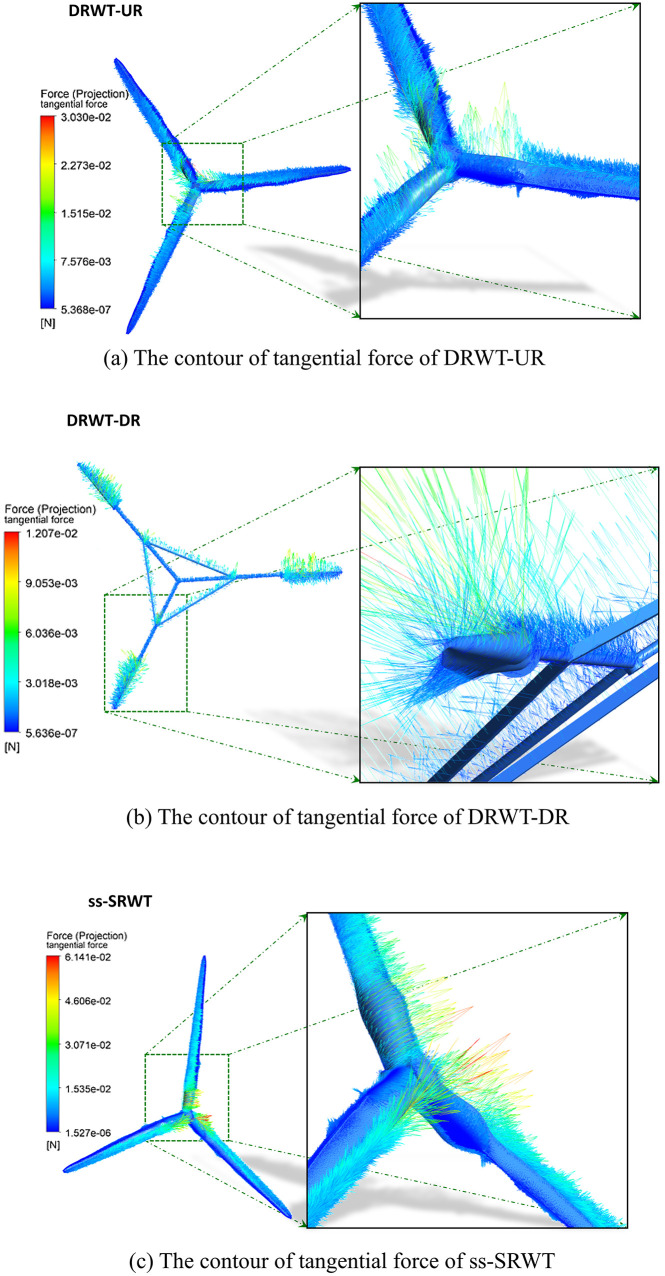
Vector contour of the tangential wall shear forces acting on the blade surfaces during operation. (Simulation parameters: U = 11.4 m/s, $Re=5\times 10^{\text{6}}$, time step Δt=0.005s).

Fig 14 illustrates the tangential shear forces on the surfaces of the blades of DRWT and ss-SRWT. The tangential shear forces on the blades of ss-SRWT are consistently higher than those on the blades of DRWT, approximately twice as much. Overall, the tangential forces experienced on the surfaces of the blades of both DRWT and ss-SRWT are significantly lower than the normal forces, only around the magnitude of $10^{\text{-}4}$ times of the normal forces.

It can be observed that the values of these two forces on the blades of DRWT are significantly lower than those on ss-SRWT. This is because, although both the DRWT and ss-SRWT have the same power and scale, the blade lengths of the two rotors in DRWT are shorter compared to ss-SRWT. This significantly improves the stress conditions of the blades during operation, indicating that the double-rotor structure of DRWT has a clear advantage in improving the stress conditions of blades.

**Fig 15:**
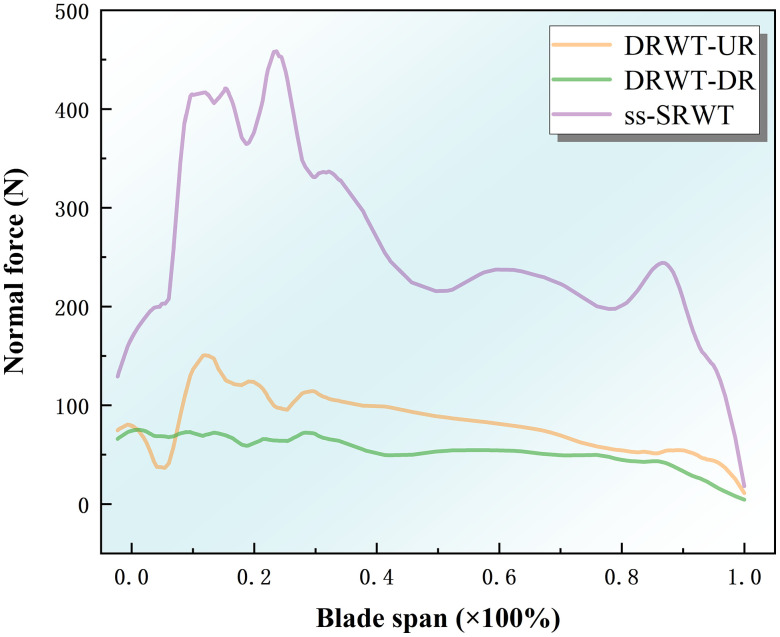
Variation curve of normal aerodynamic forces along the radial position of the blades from root to tip. Data extracted under steady operating conditions with a time step Δt=0.005s and rated wind velocity.

Fig 15 illustrates the variation of normal forces along the radial position of the blades for both DRWT and ss-SRWT. The Fig indicates that both DRWT and ss-SRWT exhibit a decreasing trend in normal forces on their blades from the root to the tip. The larger normal forces are mainly concentrated at the blade root. The average normal force on the blades of ss-SRWT is approximately 3 ~ 4 times higher than the average normal force on the blades of both rotors in DRWT.

### The results of transient structural

To ensure a clear connection between the aerodynamic analysis and the structural analysis, the aerodynamic loads obtained in Section “The results of Fluent” were transferred to the structural model as external loading conditions in the subsequent fluid-structure interaction procedure. Specifically, the pressure distribution calculated on the blade surfaces by the CFD model under the same inflow and operating conditions was mapped onto the corresponding structural mesh through the coupling interface. The distributed aerodynamic pressures were then converted into equivalent nodal loads while preserving their spatial distribution and resultant force characteristics. Based on these transferred loads, the structural responses of the blades, including stress and deformation, were further evaluated in this Section. In this way, the structural results directly reflect the mechanical effects induced by the aerodynamic loads identified in Section “The results of Fluent”.

The present fluid–structure interaction framework is a one-way coupling approach in this study. Specifically, the aerodynamic load distributions obtained from the CFD analysis in Section “The results of Fluent” are mapped onto the structural model as distributed surface loads, and the resulting structural responses, including equivalent stress and deformation, are then calculated in the structural solver. The structural deformation is not fed back to update the flow field during the current simulation cycle. Therefore, the results presented in Section “The results of Transient Structural” should be interpreted as the structural responses induced by the CFD-derived aerodynamic loads under a one-way FSI framework.

### The equivalent stress of blades

**Fig 16:**
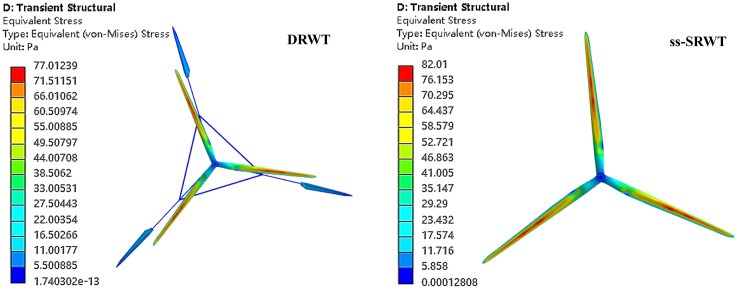
The contour of equivalent stress.

The contour of the equivalent stress on the blades of DRWT and ss-SRWT are shown in Fig 16. The distribution of the equivalent stress on the blades of DRWT-UR and ss-SRWT is similar. However, a quantitative comparison of the peak von Mises stress reveals significant differences. Under identical operating conditions, the maximum equivalent stress on the DRWT blades is 77.01 MPa, compared to 82.01 MPa for the ss-SRWT blades, corresponding to a stress reduction of approximately 6.5%. To account for uncertainty in the transient simulation, we analyzed the stress fluctuation over 5 rotation cycles. The standard deviation of the peak stress for the DRWT-UR was found to be 11.71 MPa, significantly lower than the 18.64 MPa observed for the ss-SRWT, suggesting that the DRWT structure is not only stronger but also subjected to less volatile loading conditions.

Since structural fatigue is directly driven by stress amplitude and load cycles, this significant reduction in stress fluctuation (from 18.64 MPa to 11.71 MPa) indicates that the DRWT design has the potential to substantially improve fatigue life and long-term structural reliability under continuous operation.

This paper investigated the time-varying behavior of the equivalent stress on the blades. Table 4 presents the numerical values of the equivalent stress with maximum and average of the blades of DRWT and ss-SRWT at different time instances.

**Table 4:** The equivalent stress of the blades.

Times[s]	DRWT-UR[pa]		DRWT-DR[pa]		ss-SRWT[pa]	
Times	Maximum	Average	Maximum	Average	Maximum	Average
9.7333e-004	121.61	30.266	12.371	2.168	156.09	35.304
1.9467e-003	73.073	22.641	12.544	1.9909	102.66	13.099
2.92e-003	74.031	14.615	4.1017	0.86453	72.64	30.502
3.8933e-003	77.272	20.717	1.5012	0.11118	97.453	23.77
4.8667e-003	73.959	20.301	2.777	0.37173	92.113	12.658
5.84e-003	81.445	16.99	4.4534	0.81603	84.556	16.324
6.8133e-003	71.713	21.834	6.641	1.1214	87.678	19.539
7.7867e-003	80.952	19.138	8.4806	1.4366	80.626	27.825
8.76e-003	71.102	18.956	9.5327	1.6582	87.435	21.128
9.7333e-003	79.962	21.184	11.258	1.8956	88.774	18.369
1.0707e-002	74.916	19.057	11.868	2.0533	83.447	25.769
1.168e-002	78.592	19.845	13.34	2.2701	85.388	24.803
1.2653e-002	76.649	20.703	14.218	2.4317	83.567	18.968
1.3627e-002	77.176	19.203	15.019	2.5777	83.454	22.523
1.46e-002	77.012	20.298	15.829	2.6942	82.01	20.844

**Fig 17:**
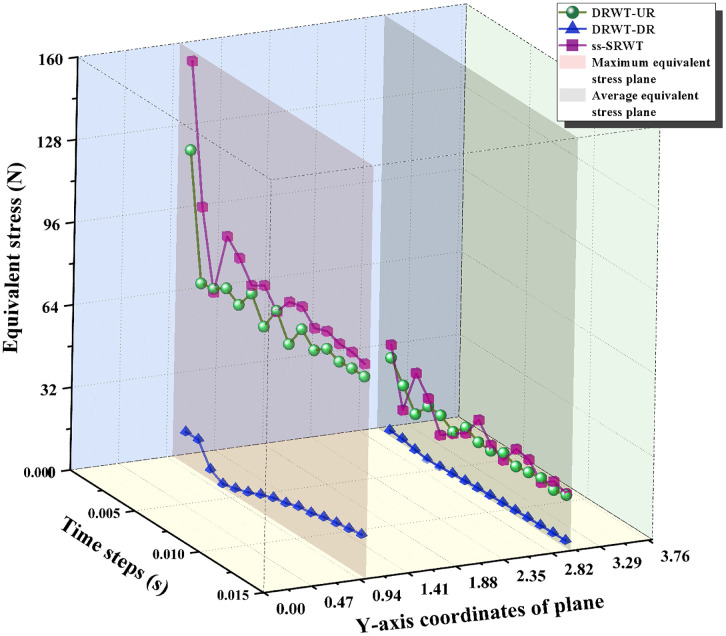
The time-varying curves of equivalent stress of blades.

Fig 17 depicts their time-varying curves. In the Fig, the curves in the red and gray planes are the maximum and average equivalent stress curves of DRWT and ss-SRWT respectively. Both the maximum and average equivalent stress of ss-SRWT is the highest, followed by DRWT-UR, although it does not decrease significantly. The equivalent stress of DRWT-DR is the lowest. Because the blades of DR are mounted on the support rods and are located mostly on the periphery of the swept areas of UR’s blades, the blades of DR do not receive the incident of the original wind when operating. Instead, they encounter a mixed wind flow after being influenced by the disturbances from UR’s blades. This avoids the direct impact of incident of income-wind on the blades of DR, resulting in the lower equivalent stress experienced by DR’s blades. Therefore, the normal force acting on the DRWT blades is also lower compared to that of the ss-SRWT, which corroborates the analysis findings presented in Section ”The results of Fluent” above. It is also evident that the structural advantages of DRWT are quite significant. However, considering the conclusion drawn in Section “The distribution of wind speed” —that the vortex structure of DRWT is more complex, with smaller but greater numbers of vortices—this would increase the load on downstream wind turbines. Therefore, this issue must be taken into account when planning the layout of DRWT wind farms.

Fig 17 also reveals that the equivalent stress on the blades of both DRWT and ss-SRWT reaches its peak during the initial startup from a stationary state, gradually stabilizing over time. This indicates that wind turbine blades experience significant stress impacts during startup, which can adversely affect their service life. To mitigate this, the design of operational control systems could incorporate blade pitch angle adjustment or braking torque control to reduce the turbine’s startup torque, thereby minimizing the startup stress.

Furthermore, it is essential to translate these raw stress reductions into operationally relevant metrics. In wind turbine engineering, component lifespan is primarily governed by fatigue caused by cyclic aerodynamic loading. The observed reduction in the standard deviation of peak stress for the DRWT-UR (11.71 MPa compared to 18.64 MPa for the ss-SRWT) indicates a substantial decrease in load amplitude volatility. Lower stress amplitudes directly correlate to reduced Damage Equivalent Loads (DELs). According to the Basquin relation for fatigue life, fatigue damage is exponentially sensitive to stress fluctuations. Therefore, even the seemingly modest 6.5% reduction in peak stress, combined with the significant 37% drop in stress standard deviation, will theoretically result in an exponential decrease in cumulative fatigue damage over time. Consequently, these stress characteristics suggest that the DRWT configuration can effectively alleviate structural fatigue, thereby extending the operational lifespan of the blades and reducing long-term maintenance costs.

### The total deformation of blades

The degree of deformation of wind turbine blades directly affects the amount of wind energy absorbed by the blades. This is because deformation alters the direction and angle of attack of the original airflow, and severe deformation can accelerate the formation of attached vortices on the blades.

**Fig 18:**
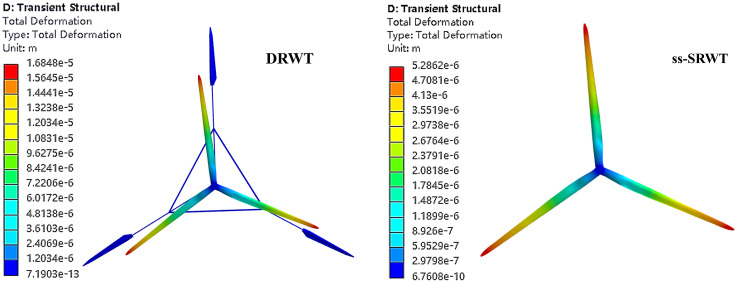
The contour of total deformation.

Fig 18 shows the contour of the total deformation on the blades of DRWT and ss-SRWT. The distribution of total deformation on the blades of DRWT-UR and ss-SRWT is similar, increasing gradually from the root to the tip of the blade. The total deformation of the DRWT-DR is minimal, but the total deformation DRWT-UR is slightly larger than that of ss-SRWT. Table 5 presents the maximum and average values of total deformation of DRWT and ss-SRWT blades at different time points.

**Table 5:** The total deformation of blades.

Times[s]		DRWT-UR[μm]		DRWT-DR[μm]		ss-SRWT[μm]
Times	Maximum	Average	Maximum	Average	Maximum	Average
9.7333e-004	7.2903e-002	2.3334e-002	4.1551e-003	2.9496e-003	1.059e-002	2.3217e-003
1.9467e-003	3.0672e-001	9.817e-002	1.8768e-002	1.3323e-002	1.7027e-001	2.7789e-002
2.92e-003	7.0531e-001	2.2575e-001	4.4161e-002	3.1349e-002	2.5993e-001	5.7682e-002
3.8933e-003	1.247	3.9914e-001	7.5494e-002	5.3591e-002	3.6642e-001	8.1329e-002
4.8667e-003	1.9301	6.1777e-001	1.0803e-001	7.6684e-002	4.8987e-001	1.0874e-001
5.84e-003	2.7575	8.8258e-001	1.3834e-001	9.8202e-002	6.3058e-001	1.3997e-001
6.8133e-003	3.7305	1.194	1.6379e-001	1.1627e-001	7.8871e-001	1.7508e-001
7.7867e-003	4.8506	1.5525	1.8223e-001	1.2936e-001	9.643e-001	2.1406e-001
8.76e-003	6.1184	1.9583	1.9184e-001	1.3618e-001	2.1046	4.6721e-001
9.7333e-003	7.5343	2.4115	1.9107e-001	1.3564e-001	2.3859	5.2966e-001
1.0707e-002	2.893e-001	9.0987	1.7858e-001	1.2677e-001	3.0026	6.6657e-001
1.168e-002	3.4378e-001	10.812	1.5315e-001	1.0872e-001	3.3381	7.4104e-001
1.2653e-002	4.03e-001	12.675	1.1362e-007	8.0654e-002	3.6916	8.1953e-001
1.3627e-002	4.6697e-001	14.687	5.8907e-002	4.1816e-002	4.0633	9.0204e-001
1.46e-002	5.357e-001	16.848	1.1874e-002	8.4293e-003	5.2862	1.1735

**Fig 19:**
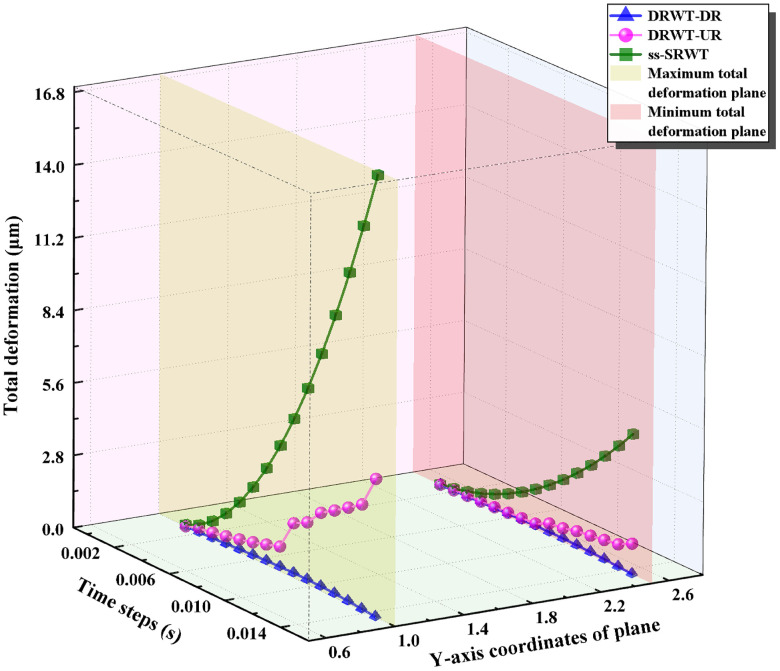
The time-varying curve of total deformation.

Fig 19 depicts the relationship between their total deformation and time. In the Fig, the curves in the yellow and red planes are the maximum and average total deformation curves of DRWT and ss-SRWT respectively. The total deformation of DRWT-UR blades shows a noticeable increase over time, while the total deformation of DRWT-DR and ss-SRWT blades exhibits a relatively stable temporal trend. It Should be noted that for total deformation, the value is highest for DRWT-UR, followed by ss-SRWT, and DRWT-DR has the smallest total deformation. This differs from the conclusions drawn regarding aerodynamic forces and equivalent stress in the aforementioned observation. However, since the overall deformation amount is approximately less than $10^{-5}$ of the length of the blade, it has almost no impact on the performance of the blades during operation.

### Sensitivity analysis

**Fig 20:**
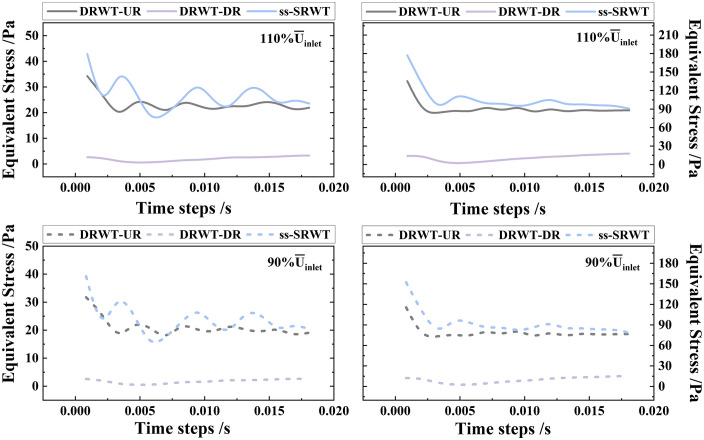
The equivalent stress after a 10% increase or decrease in the average inlet stochastic wind speed.

To verify that the observed performance differences between the DRWT and ss-SRWT are robust and not merely artifacts of specific parameter tuning, a sensitivity analysis was conducted. A key systemic parameters were subjected to reasonable variations: average inlet stochastic wind speed (±10%). The results are presented in Fig 20.

The sensitivity results indicate that under all varied conditions, the DRWT consistently outperformed the ss-SRWT. Specifically, the reduction in peak blade equivalent stress for the DRWT compared to the ss-SRWT fluctuated narrowly between 30.98% (110% ${\overset{―}{U}}_{inlet}$) and 31.52% (90% ${\overset{―}{U}}_{inlet}$), while the baseline was 28.35% (${\overset{―}{U}}_{inlet}$), with the stress standard deviation remaining consistently lower. These findings confirm that the superior aerodynamic efficiency and mechanical robustness of the DRWT are inherent to its differential double-rotor structural design and are robust to reasonable variations in mass, inertia, and environmental boundary conditions.

## Conclusions

In this study, the aerodynamic and mechanical performances of a novel Double-Rotor Wind Turbine (DRWT) featuring a differential planetary gearbox were investigated and compared with a geometrically equivalent Single-Rotor Wind Turbine (ss-SRWT). Our findings reveal that the DRWT exhibits superior aerodynamic efficiency and mechanical robustness compared to its single-rotor counterpart, evidenced by a 4.2% faster wake velocity recovery and a 28.35% decrease in structural stress loads. The following conclusions can be drawn:

(I) Aerodynamic Performance: The simulation results indicate that the DRWT configuration has the potential to achieve higher wind energy capture efficiency compared to the ss-SRWT. The interaction between the upwind and downwind rotors creates a complex wake structure that, under the simulated conditions, appears to contribute to enhanced power generation capabilities.
(II) Mechanical Performance: The structural analysis confirms that the DRWT design offers significant mechanical advantages that translate into operationally relevant metrics. Under stochastic wind loads, the DRWT not only exhibited lower peak stresses but, more importantly, demonstrated a significantly reduced stress variance. This mitigation of load volatility implies a substantial reduction in Damage Equivalent Loads (DELs). Consequently, the proposed structural and transmission design is highly conducive to minimizing cumulative fatigue damage, thereby offering a reliable pathway to extending the fatigue life and overall service duration of both the blades and drivetrain components relative to traditional single-rotor designs.

However, it is essential to acknowledge the limitations inherent in this purely numerical investigation. Specifically, while the current FSI analysis demonstrates reduced peak stresses and load volatility, it does not fully address the cumulative fatigue, high-cycle load effects, and long-term structural reliability over a turbine’s lifespan, which are critical for engineering practice. To fully validate the practical feasibility and superiority of the proposed DRWT, future work must incorporate comprehensive fatigue life assessments (e.g., using Palmgren-Miner damage rules), long-term dynamic load evaluations under varied turbulent wind conditions, and experimental validations, such as scaled wind tunnel testing or field prototype trials, is critical to corroborate the simulation outcomes and to assess the impact of real-world factors—such as manufacturing tolerances and complex environmental turbulence—that were not fully captured in this numerical study.

Furthermore, while this study provides preliminary insights into the aerodynamic performance to provide dynamic loads for the structural solver, the discussion of wake behavior—particularly the complex wake–wake interactions inherent in DRWT configurations—remains relatively fundamental. A rigorous quantitative analysis of wake velocity deficit, turbulence intensity, and spectral characteristics falls primarily into the domain of pure computational fluid dynamics (CFD). Because the primary focus of this paper is to validate the structural and load-optimization advantages of the DRWT via FSI analysis, an exhaustive aerodynamic investigation is beyond the current scope. These issues will be investigated in a forthcoming study using a coupled double-rotor wake model.
